# The multifaceted roles of BAF60 subunits in muscle: regulation of differentiation, reprogramming, and metabolic homeostasis

**DOI:** 10.3389/fcell.2025.1594423

**Published:** 2025-06-26

**Authors:** Yaoxia Liu, Zhen Fan, Yang Zhai, Haotian Huang, Ruifeng Shi, Tao Wang

**Affiliations:** ^1^ Department of Geriatric Endocrinology, Sichuan Provincial People’s Hospital, School of medicine, University of Electronic Science and Technology of China, Chengdu, China; ^2^ Department of Pediatrics, West China Second University Hospital, Sichuan University, Chengdu, China; ^3^ Chengdu Women’s and Children’s Central Hospital, Chengdu, China; ^4^ School of Medicine, University of Electronic Science and Technology of China, Chengdu, China; ^5^ Sichuan Provincial Center for Mental Health, Sichuan Provincial People’s Hospital, School of Medicine, University of Electronic Science and Technology of China, Chengdu, China; ^6^ Key Laboratory of Birth Defects and Related Diseases of Women and Children (Sichuan University), Ministry of Education, Chengdu, China

**Keywords:** BAF60 subunit, BAF60a, BAF60b, Baf60c, muscle, differentiation, reprogramming, metabolic homeostasis

## Abstract

Muscle development and metabolic homeostasis are critical aspects of human health. As a key component of the SWI/SNF chromatin remodeling complex, the BAF60 subunit plays a widespread and crucial role in the differentiation, reprogramming, and metabolic regulation of skeletal, cardiac, and smooth muscle. Recent studies reveal that BAF60c promotes skeletal muscle differentiation and regeneration by interacting with MyoD, while also driving cardiac-specific gene expression and cooperating with transcription factors such as NKX2.5 and GATA Binding Protein 4 (GATA4) during heart development and remodeling. In smooth muscle, BAF60c interacts with serum response factor to maintain contractility and homeostasis by reducing inflammation and apoptosis. In contrast, BAF60a promotes inflammatory responses and extracellular matrix degradation, contributing to vascular diseases such as abdominal aortic aneurysm and atherosclerosis. Importantly, different BAF60 isoforms exhibit antagonistic, synergistic, or mutually exclusive effects in different muscle types, highlighting their complexity. This review provides a comprehensive overview of BAF60 isoforms’ regulatory roles, with an emphasis on their potential as therapeutic targets for muscle-related metabolic and vascular diseases.

## Introduction

Chromatin, the carrier of genetic material, is composed of DNA packaged into nucleosomes. Adenosine triphosphate (ATP)-dependent chromatin remodeling complexes utilize ATPase activity to regulate chromatin structure, making chromatin more accessible and controlling gene transcription (Cairns, 2009). Among these complexes, Switch/Sucrose Non-Fermentable (SWI/SNF) plays a key role in regulating gene expression across tissues, with its functional diversity driven by variations in subunit composition (Puri and Mercola, 2012; Kadoch and Crabtree, 2015; Wang et al., 2018). Within the SWI/SNF complex, Brg1/BRM-associated factor 60 (BAF60) subunits are unique and essential. Acting as a bridge between the core complex and transcription factors, BAF60 subunits recruit SWI/SNF to specific gene promoters, regulating transcription (Collingwood et al., 1999; Fry and Peterson, 2001; Aras et al., 2019). The three isoforms—BAF60a, BAF60b, and BAF60c—perform distinct physiological roles in a tissue-specific manner. BAF60c, encoded by the *Smarcd3* gene, is predominantly expressed in muscle, brain, and heart tissues. It serves as a critical regulatory factor for the development of the heart, retina, and skeletal muscle (Meng et al., 2017). BAF60c promotes glycolysis in both cardiac and skeletal muscles and enhances the transcription of hepatic lipogenesis-related genes (Wang et al., 2013; Zhang et al., 2016). BAF60a (Smarcd1), is primarily expressed in the liver, brain, adipose tissue, and skeletal muscle. It facilitates hepatic fatty acid β-oxidation, improves fatty liver, and maintains cholesterol homeostasis (Wang et al., 2018; Zhang et al., 2016). BAF60b (Smarcd2), is highly expressed in immune cells and acts as a key regulatory factor for granulocyte development and function (Witzel et al., 2017; Liu et al., 2024).

The BAF60 subunits, particularly BAF60c, are emerging as central regulators of tissue-specific transcriptional programs. Rather than being identified through a single systematic interactome study, BAF60c′s interactions with lineage-determining transcription factors have been revealed across different muscle types in a tissue-specific and context-dependent manner. In this review, we focus on the roles of BAF60 isoforms in muscle biology, including their involvement in differentiation, reprogramming, regeneration, and metabolic regulation. Despite significant advances, the mechanisms underlying their functions remain incompletely understood. The intricate interplay among BAF60 isoforms often results in antagonistic, synergistic, or tissue-specific effects, underscoring their complexity and functional versatility.

Recent studies have demonstrated that BAF60 subunits play critical regulatory roles in skeletal, cardiac, and smooth muscle. These tissues, although distinct in function, share a reliance on intricate genetic and epigenetic mechanisms for their development, reprogramming, and metabolic homeostasis. Specifically, BAF60c interacts with muscle-specific transcription factors, such as myogenic differentiation 1 (MyoD) in skeletal muscle, NK2 homeobox 5 (NKX2.5) in cardiac muscle, and serum response factor (SRF) in smooth muscle. These interactions orchestrate chromatin remodeling and activate key gene networks.

This review integrates recent findings on the multifaceted roles of BAF60 subunits across different muscle types, including their involvement in differentiation, regeneration, and metabolic regulation. By exploring these mechanisms, we aim to provide novel insights into their therapeutic potential for muscle-related diseases, bridging fundamental research and translational applications.

## BAF60c determines the fate of myogenic differentiation

During embryonic development, the differentiation of trunk- and limb-derived skeletal muscles begins with multipotent mesodermal precursor cells, which, under myogenic signals, differentiate into myogenic progenitor cells (MPCs) expressing Pax3 and Pax7 (Buckingham and Rigby, 2014). These MPCs subsequently form myoblasts that proliferate, fuse, and mature into functional multinucleated myofibers, establishing skeletal muscle tissue (Li et al., 2018). The differentiation of skeletal muscle is regulated by BAF60c and the myogenic regulatory factor family (MRFs), among which MyoD plays a pivotal role in muscle growth and development (MacQuarrie et al., 2013; Toto et al., 2016). BAF60c interacts with MyoD, binding to the E-box sequence (CANNTG) in muscle-specific gene promoters (Toto et al., 2016). In response to the myogenic signal p38 mitogen-activated protein kinase (p38 MAPK), BAF60c is phosphorylated and, together with other pioneer factors, recruits the Brahma-related gene 1 (Brg1) core subunit to chromatin (Simone et al., 2004; Forcales et al., 2012). This results in the formation of a MyoD–BAF60c–Brg1 SWI/SNF complex in myoblasts under differentiation-inducing conditions and in satellite cells responding to regenerative cues, which remodels chromatin, disrupts nucleosomes, activates muscle-specific gene expression, and promotes the myogenic differentiation of MPCs (Simone et al., 2004; Forcales et al., 2012) (Table 1).

**TABLE 1:** Roles and mechanisms of BAF60 subunits in skeletal muscle differentiation.

Muscle type	Complex condition	Binding regions	Cell types	Upstream factors		BAF60 roles
				Upregulation	Downregulation	
Skeletal muscle	MyoD + BAF60c + Brg1 (Simone et al., 2004; Forcales et al., 2012)	CANNTG box (Toto et al., 2016)	myoblasts (Simone et al., 2004; Forcales et al., 2012)	P38 (Lee et al., 2016b); PKN2 (Lee et al., 2016a)	SOD1G93A (Martini et al., 2015)	Antagonistic Role: BAF60c promotes myogenic differentiation; BAF60a/b inhibit myogenic differentiation
			C2C12 cell (Lickert et al., 2004)	Bakuchiol (Lee et al., 2016b)	HDAC4 (Martini et al., 2015)	
			Skeletal muscle satellite cells (Simone et al., 2004)	RNAlin-RAM (Yu et al., 2017)		

This table summarizes the interactions and regulatory mechanisms by which BAF60 isoforms influence skeletal muscle differentiation, including antagonistic functions between BAF60c and BAF60a/b. References in square brackets correspond to studies supporting the respective findings and mechanisms.

P38, p38 Mitogen-Activated Protein Kinase; PKN2, Protein Kinase N2; RNAlin-RAM, RNA-Linked Regulator of Myogenesis; SOD1G93A, Superoxide Dismutase 1, G93A Mutation; HDAC4, Histone Deacetylase 4; C2C12, mouse myoblast cell line.

Silencing BAF60c expression in mouse embryos and C2C12 cells (a mouse myoblast cell line commonly used to study skeletal muscle differentiation and myogenesis) impedes myogenic differentiation. RNAi-mediated silencing of BAF60c in mouse embryos results in abnormal skeletal muscle differentiation (Lickert et al., 2004). In C2C12 cells, downregulation of BAF60c by RNAi prevents MyoD from recruiting chromatin, blocking the expression of hundreds of myogenic genes and disrupting myogenic differentiation (Forcales, 2012). Embryonic stem cells (ESCs) lack BAF60c expression, which is essential for the formation of the MyoD–BAF60c–Brg1 SWI/SNF complex that facilitates chromatin remodeling and gene activation during myogenic differentiation. As a result, even ectopic expression of MyoD in ESCs is insufficient to induce myogenic differentiation (Albini et al., 2013). MyoD alone cannot effectively bind to muscle-specific gene promoters in ESCs due to their tightly packed chromatin structure. However, forced expression of BAF60c enables MyoD to interact with the SWI/SNF complex containing BAF60c, leading to chromatin remodeling, activation of muscle-specific gene expression, and the production of contractile myosin in ESCs (Albini and Puri, 2014). This highlights the crucial role of BAF60c in enabling MyoD to overcome the epigenetic barriers present in ESCs and activate the myogenic program. Additionally, skeletal muscle cells can be generated from Duchenne muscular dystrophy patient-derived human induced pluripotent stem cells (hiPSCs) by inducing MyoD and BAF60c expression (Caputo et al., 2020) (Table 2). This model is crucial for studying the pathophysiology of DMD, offering insights into muscle degeneration and regeneration. It also facilitates testing therapeutic strategies, such as gene editing and drug screening, for developing effective treatments.

**TABLE 2:** Roles of BAF60c subunits in skeletal muscle reprogramming.

Muscle type	Reprogramming		
	Cell type	Transcription factors	Outcome
Skeletal muscle	ESCs (Albini et al., 2013)	MyoD + BAF60c (Albini et al., 2013; Caputo et al., 2020; Saccone et al., 2014)	Skeletal muscle cells with contractile myosin (Albini et al., 2013)]
	hiPSCs of DMD patient (Caputo et al., 2020)		Skeletal muscle cells (Caputo et al., 2020)
	FAPs of young mdx mice (Saccone et al., 2014)		Myogenic differentiation (Saccone et al., 2014)

This table illustrates how BAF60c, often in combination with MyoD, facilitates the reprogramming of stem cells or progenitors into skeletal muscle cells. References in square brackets correspond to studies supporting the respective findings and mechanisms.

ESCs, Embryonic Stem Cells; hiPSCs, Human Induced Pluripotent Stem Cells; FAPs, Fibro-Adipogenic Progenitor Cells; DMD, duchenne muscular dystrophy.

## BAF60c modulates skeletal muscle regeneration

Skeletal muscle satellite cells, originating from MPCs during embryonic development, remain quiescent in mature muscle fibers but are activated upon injury or stress to proliferate and differentiate into myoblasts, facilitating muscle repair and regeneration (Bharathy et al., 2013). In muscle satellite cells, the formation of a complex between MyoD and BAF60c can also be detected (Forcales et al., 2012). When these cells are exposed to regeneration signals such as p38 kinase induction, BAF60c is phosphorylated and activated, which subsequently recruits Brg1 to specific chromatin regions. This leads to the formation of the SWI/SNF complex, which activates the transcription of genes related to myogenic differentiation (Forcales et al., 2012). Linc-RAM has been shown to promote the formation of the MyoD–BAF60c–Brg1 complex. In Linc-RAM knockout mouse models, defects in satellite cell differentiation and delayed muscle regeneration have been observed (Yu et al., 2017) (Table 1).

**FIGURE 1:**
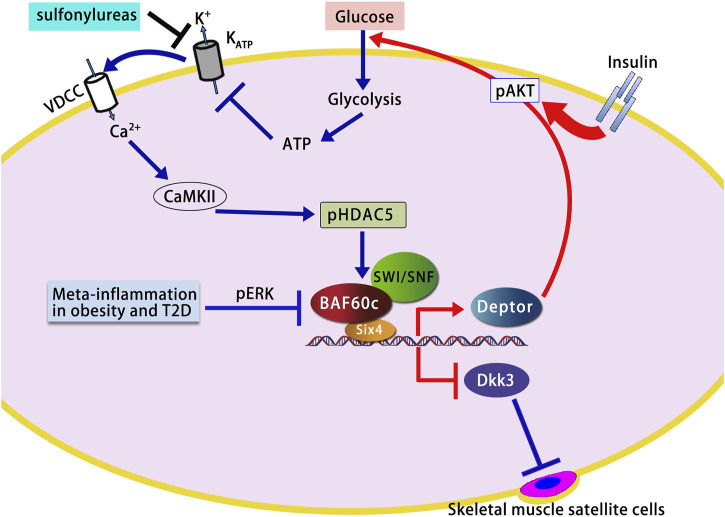
Role of BAF60c in skeletal muscle glucose metabolism and regeneration. BAF60c promotes glycolytic metabolism and muscle regeneration by activating the Deptor–AKT signaling pathway and suppressing Dkk3-mediated inhibitory signals. Its function is regulated by glucose sensing, sulfonylurea action, and ERK signaling in metabolic disease states. The figure is based on illustrations from Wang et al. (2018) and Meng et al. (2017), with modifications to incorporate additional pathways and findings relevant to this study. VDCC, Voltage-Dependent Calcium Channel; KATP, ATP-Sensitive Potassium Channel; CaMKII, Calcium/Calmodulin-Dependent Protein Kinase II; HDAC5, Histone Deacetylase 5; SWI/SNF, SWItch/Sucrose Non-Fermentable; AKT, Protein Kinase B; Deptor, DEP Domain-Containing MTOR-Interacting Protein; ERK, Extracellular Signal-Regulated Kinase; Dkk3, Dickkopf-3; T2D, Type 2 Diabetes; ECM, Extracellular Matrix.

Xu et al. (2023) constructed mature muscle fiber-specific Baf60c knockout (BcMKO), Baf60c overexpression, and adult muscle satellite cell-specific Baf60c knockout (BcSCKO) mouse models, providing new insights into the role of Baf60c in muscle regeneration. The results demonstrated that BcMKO inhibited muscle regeneration and reduced contractile function, while Baf60c overexpression promoted muscle regeneration. In contrast, in BcSCKO mice, the expression of several differentiation-related genes, including *Myomaker* and *MyoG*, was significantly downregulated in muscle tissues. However, no overt alterations in muscle structure or fiber size distribution were observed. This apparent discrepancy suggests that while Baf60c in muscle stem cells modulates myogenic gene expression, its inactivation alone is insufficient to cause immediate structural changes, potentially due to functional redundancy or compensatory mechanisms from surrounding niche cells or residual Baf60c-independent pathways. Alternatively, the transcriptional changes may precede morphological alterations and primarily impact regenerative capacity under stress or injury rather than baseline muscle maintenance (Xu et al., 2023). Further investigation revealed that Baf60c interacts with the transcription factor Sine oculis homeobox homolog 4 (Six4) to suppress the expression of the muscle-secreted factor Dickkopf-related protein 3 (Dkk3), which otherwise inhibits satellite cell differentiation. By suppressing Dkk3, Baf60c indirectly promotes satellite cell function and muscle regeneration. Importantly, Baf60c expression is downregulated in obese and type 2 diabetic patients, leading to elevated levels of Dkk3, which may contribute to muscle regeneration dysfunction in these populations (Xu et al., 2023) (Figure 1).

## Multilayer regulation of BAF60c expression and activity during skeletal muscle differentiation

BAF60c plays a crucial role in skeletal muscle differentiation by forming the MyoD–BAF60c–Brg1 SWI/SNF chromatin remodeling complex. Its regulation involves transcriptional control, post-translational modification, and dynamic complex assembly. At the transcriptional level, inflammatory cytokines such as Tumor Necrosis Factor Alpha (TNF-α) repress BAF60c (Smarcd3) expression via NF-κB–associated epigenetic mechanisms, including reduced Histone 3, Lysine 4 (H3K4) trimethylation and increased H3K9me2 at its promoter. This repression has been observed in both C2C12 cells and primary human myotubes and contributes to impaired muscle metabolic adaptation in obese and diabetic conditions (Meng et al., 2013). In contrast, several signaling pathways influence BAF60c function and recruitment without directly altering its expression. Protein kinase C-related kinase 2 (PKN2) activates AKT signaling and enhances the recruitment of BAF60c and MyoD to myogenic promoters, thereby promoting myoblast differentiation. Overexpression of PKN2 accelerates myogenic progression, whereas its depletion impairs differentiation in C2C12 cells (Lee et al., 2016a). Similarly, bakuchiol, a plant-derived compound, activates the p38 MAPK pathway and enhances BAF60c recruitment to the Myogenin promoter via phosphorylation, facilitating the MyoD–BAF60c interaction and promoting muscle gene activation (Lee et al., 2016b). Furthermore, the long non-coding RNA Linc-RAM directly interacts with MyoD in the nucleus and facilitates the assembly of the MyoD–BAF60c–Brg1 complex. Knockout of Linc-RAM in mice leads to delayed muscle regeneration and defective satellite cell differentiation, highlighting the *in vivo* relevance of this regulatory mechanism (Yu et al., 2017). Additionally, expression of the mutant SOD1G93A gene—an ALS-associated variant of the superoxide dismutase 1 (SOD1) gene—impairs BAF60c-mediated myogenesis by upregulating HDAC4 and suppressing the Akt/p70S6K and MAPK signaling pathways, thereby disrupting the BAF60c–SWI/SNF complex and downregulating key myogenic regulators (Martini et al., 2015). Treatment with the HDAC II inhibitor trichostatin A (TSA) partially restores myogenic gene expression and myotube formation in C2C12 cells (Martini et al., 2015). Although these regulatory mechanisms were initially studied independently, they converge functionally to control the availability, activity, and recruitment of BAF60c to chromatin, ensuring precise execution of the myogenic program in both cultured cells and regenerating muscle tissue.

## Antagonistic roles of BAF60c and BAF60a/b in skeletal muscle differentiation

BAF60c cooperates with MyoD to recruit the SWI/SNF complex to myogenic promoters, thereby facilitating the activation of myogenic gene transcription. In contrast, BAF60a and BAF60b competitively inhibit this process, fine-tuning the balance between differentiation and quiescence. Studies in chick embryos revealed that during myogenic differentiation, BAF60c expression increases, whereas BAF60a and BAF60b levels decrease. Knockdown of Baf60c or overexpression of Baf60a/b inhibits muscle differentiation. In addition, muscle-specific microRNAs (miR-1/206 and miR-133) downregulate BAF60a/b, thereby facilitating BAF60c incorporation into the Brg1 complex and enhancing myogenic gene transcription (Goljanek-Whysall et al., 2014). Fibro-adipogenic progenitor cells (FAPs), located in adult skeletal muscle stroma, are multipotent mesenchymal cells capable of differentiating into adipocytes or fibroblasts and play crucial roles in muscle regeneration and homeostasis (Chen W. et al., 2022). In young *mdx* mice, a Duchenne muscular dystrophy model, HDAC inhibitors (HDACis) enhance MyoD and BAF60c expression, upregulate myogenic microRNAs (e.g., miR-1, miR-133, and miR-206), and suppress BAF60a/b, thereby promoting the myogenic conversion of FAPs while inhibiting adipogenic and fibrogenic fates (Saccone et al., 2014). To explore the *in vivo* roles of different BAF60 isoforms, Meng et al. generated muscle-specific knockout models for BAF60a (AKO), BAF60c (CKO), and the double knockout (ACKO) (Meng et al., 2018). Under basal conditions, neither AKO nor CKO alone altered muscle mass, indicating partial functional redundancy between these isoforms (Meng et al., 2018). However, the ACKO phenotype exhibited impaired glycolytic and oxidative metabolism, resembling that of CKO mice, but with further reduced metabolic capacity, suggesting that BAF60a cannot compensate for BAF60c loss in regulating muscle bioenergetics and energy homeostasis (Meng et al., 2018). While BAF60c promotes and BAF60a suppresses myogenic differentiation, single-knockout studies show that deletion of either gene alone does not disrupt muscle mass, likely due to compensatory mechanisms. However, double deletion still mimics BAF60c loss, underscoring a nonlinear, context-dependent interplay between BAF60 isoforms: their antagonism is functionally significant during early differentiation, but in mature muscle, their roles may be buffered by additional pathways.

## BAF60c regulates glucose metabolism in skeletal muscle

Skeletal muscle fibers are classified into types I, IIa, IIx, and IIb based on their myosin heavy chain isoform composition and metabolic profiles. Type I and IIb fibers represent the oxidative and glycolytic extremes, respectively, while type IIa and IIx display intermediate properties along a metabolic continuum (Duan et al., 2017). Hybrid fibers, which co-express multiple myosin isoforms, are also common, especially during muscle plasticity or development. Notably, type IIb fibers are absent in human skeletal muscles and are restricted to rodents (Duan et al., 2017). Meng et al. (2013) reported that BAF60c is highly expressed in fast/glycolytic type IIb fibers in mice. Muscle-specific overexpression of BAF60c induced a metabolic shift in skeletal muscle from oxidative to glycolytic metabolism, as evidenced by SDH and α-GPDH histochemical staining, altered gene expression of metabolic markers, and increased lactate production. Conversely, muscle-specific deletion of BAF60c reduced glycolytic capacity and impaired exercise endurance. Mechanistically, this metabolic reprogramming is mediated through a transcriptional complex formed by BAF60c and Six4, which recruits the SWI/SNF complex to the promoter of Deptor—a DEP domain-containing mTOR-interacting protein—thereby enhancing Deptor expression and activating AKT signaling to promote glycolytic gene expression (Meng et al., 2013). Deptor promotes AKT activation in an insulin-independent manner, enhancing glucose uptake by skeletal muscle and promoting glycolysis (Wang et al., 2018; Meng et al., 2017). In skeletal muscle cells, physiological concentrations of glucose induce the expression of BAF60c and Deptor and stimulate AKT activation, whereas fatty acids and amino acids do not have this effect (Wang et al., 2018; Meng et al., 2017). Further research indicates that ATP generated by glucose metabolism triggers a Ca^2+^ response through ATP-sensitive potassium (KATP) channels, activating Calcium/Calmodulin-dependent protein kinase II (CaMKII), which phosphorylates Histone deacetylase 5 (HDAC5), leading to increased expression of BAF60c and Deptor and subsequent AKT activation (Wang et al., 2018; Meng et al., 2017). This glucose sensing pathway works in synergy with the insulin signaling pathway to maintain glucose homeostasis. Additionally, sulfonylureas (a class of oral anti-diabetic drugs that stimulate insulin secretion from pancreatic β-cells to reduce blood glucose levels) activate the BAF60c-Deptor-AKT pathway by inhibiting KATP channels, thereby lowering blood glucose (Meng et al., 2017) (Figure 1). Importantly, recent findings have revealed that the glucose-lowering effects of sulfonylureas are not limited to pancreatic β-cells but also involve direct actions on skeletal muscle. Specifically, sulfonylureas trigger calcium signaling in myocytes via KATP channel inhibition, leading to the induction of BAF60c and Deptor expression, and subsequent AKT activation. This insulin-independent pathway enhances glucose uptake and glycolytic metabolism in skeletal muscle, contributing to systemic glucose homeostasis. These findings suggest that the BAF60c-Deptor-AKT axis in skeletal muscle represents a critical extrapancreatic target of sulfonylureas and may offer new therapeutic opportunities for treating metabolic disorders such as type 2 diabetes (Meng et al., 2017).

In obesity, elevated TNF-α levels suppress the expression of BAF60c (Meng et al., 2013). Studies have found that in diabetic mice, the expression of BAF60c and Deptor is downregulated due to activation of the Extracellular signal-regulated kinase (ERK) pathway by meta-inflammation (Wang et al., 2018; Meng et al., 2013). Rescue of this pathway by either treatment with an ERK inhibitor or transgenic expression of BAF60c in skeletal muscle ameliorates insulin resistance and improves whole-body glucose homeostasis in High-fat diet (HFD)-induced or genetically obese mice (Wang et al., 2018; Meng et al., 2013) (Figure 1).

Meng et al.’s further research revealed that BAF60a and BAF60c exhibit functional redundancy and complementarity in overall metabolic regulation (Meng et al., 2018; Meng et al., 2014). In HFD-induced obese mice, the expression levels of BAF60c protein and mRNA were significantly reduced, whereas BAF60a mRNA levels remained unchanged (Meng et al., 2014). By generating AKO, CKO, and ACKO mouse models, microarray analysis revealed that the transcriptional profile of ACKO mice closely resembled that of CKO mice, indicating that BAF60a is unable to compensate for the transcriptional programs regulated by BAF60c in skeletal muscle (Meng et al., 2018). While there was no significant difference in exercise endurance between AKO/CKO and wild-type mice, ACKO mice showed a 35% decline in exercise performance, with significant changes in blood glucose and lactate levels, indicating redundancy in the roles of BAF60a and BAF60c in muscle metabolism. ACKO mice exhibited significant resistance to HFD-induced obesity, with improved insulin sensitivity and glucose tolerance. Further mechanistic studies revealed that inactivation of BAF60a and BAF60c enhances energy expenditure through the activation of calcium cycling and thermogenesis, improving metabolic parameters, and promoting Insulin-like growth factor II (IGF2) expression (Meng et al., 2018). Notably, previous studies have shown that elevated circulating IGF-II levels can improve metabolic parameters, including reducing obesity and enhancing glucose levels (Da Costa et al., 1994; Rossetti et al., 1996).

## BAF60c is a core factor in cardiac differentiation and development

The development of the heart begins with multipotent cardiac progenitor cells (CPCs) derived from the mesoderm. These cells, regulated by signaling cues, differentiate into myocardial precursor cells, which then form primordial cardiomyocytes and construct the primary heart tube (Gittenberger-de Groot et al., 2013; Williams et al., 2019). As the cells proliferate and migrate, the heart tube folds and expands, gradually acquiring the ability to pump blood, and eventually differentiates into the atria and ventricles, completing the development of all functional regions of the heart (Gittenberger-de Groot et al., 2013; Williams et al., 2019). During early cardiomyocyte differentiation, transcription factors such as NKX2-5, PITX2, GATA4, and TBX5 orchestrate cardiac lineage specification and heart tube formation. Later activation of genes like HAND2 (Heart and Neural Crest Derivatives Expressed 2) and sarcomeric genes such as MYH6 (α-Myosin Heavy Chain) and MYH7 (β-Myosin Heavy Chain) support chamber maturation and contractile function. These processes are modulated by chromatin remodelers (e.g., BAF60c), structural proteins (e.g., XIN (X-Linked Inhibitor of NF-κB)), and key growth factors such as VEGF (Vascular Endothelial Growth Factor), FGF (Fibroblast Growth Factor), and PDGF (Platelet-Derived Growth Factor). Disruptions in these gene regulatory networks are associated with congenital heart defects (CHDs), including septal defects and aortic malformations (Williams et al., 2019) (Table 3).

**TABLE 3:** Roles of BAF60 subunits in cardiac muscle development.

Muscle type	Complex condition	Binding regions	Cell types	Upstream factors		BAF60 roles
				Upregulation	Downregulation	
Cardiac muscle	BAF60C + GATA 4+NKX2.5+TBX5+ Brg1 (Lickert et al., 2004)	Heart-specific enhancers (Lickert et al., 2004)	Cardiac progenitor cells (Al-Maqtari et al., 2017)	Isl1 (Gao et al., 2019)	Cerl2 (by inhibiting TGFβ/Nodal) (Al-Maqtari et al., 2017)	The cooperative role: BAF60c cooperates with BAF60a/b/c to promote cardiac-specific gene expression and differentiation
			Cardiomyocytes (Chen et al., 2012)	Wnt/β-catenin signaling pathway (Klaus et al., 2012)		
			HeLa cell (Lickert et al., 2004)	Cerberus-1 (by inhibiting Nodal and BMP signaling) (Cai et al., 2013)		
				Apj (by activating Cerberus-1) (D'Aniello et al., 2013)		

This table outlines the cooperative interactions of BAF60c with cardiac transcription factors during embryonic heart development and cardiomyocyte lineage specification. References in square brackets correspond to studies supporting the respective findings and mechanisms.

Isl1, ISL; LIM, Homeobox 1; Wnt/β-catenin, Wnt/Beta-Catenin Signaling Pathway; Cerberus-1, Cerberus 1; Apj, Apelin Receptor; Cerl2, Cerberus-like 2; Brg1, Brahma-related gene 1.

BAF60c is highly expressed in the early stages of mouse heart development, particularly in the heart and somites, as shown by Lickert et al. and others. BAF60c is highly expressed during early mouse heart development, particularly in the heart and somites, as shown in multiple models including mice and axolotls (Lickert et al., 2004; Nakamura et al., 2016). This early expression is essential for cardiomyocyte proliferation and morphogenesis, as demonstrated by siRNA knockdown studies, which resulted in impaired cardiac development, structural defects, and reduced cardiomyocyte proliferation (Lickert et al., 2004; Nakamura et al., 2016). Moreover, BAF60c expression is upregulated upon cardiac injury, correlating with increased regeneration and proliferation (Nakamura et al., 2016). Constitutive knockout of Baf60c in mouse embryos leads to cardiac hypoplasia, growth impairment, and dysfunction, resulting in embryonic lethality (Sun et al., 2018). Conditional deletion of Baf60c in cardiomyocytes causes postnatal dilated cardiomyopathy with chamber dilation, ventricular wall thinning, and reduced contractile function (Sun et al., 2018). Together, these findings highlight the indispensable role of BAF60c in regulating cardiomyocyte differentiation and heart development.

BAF60c facilitates the formation of transcriptional complexes by bridging cardiac-specific transcription factors—GATA4, NKX2.5, and T-Box Transcription Factor 5 (TBX5)—with the Brg1 ATPase of the SWI/SNF chromatin remodeling complex. This function has been demonstrated across multiple experimental systems. Immunoprecipitation experiments and co-expression assays in HeLa cells (a human cervical cancer cell line widely used in cancer and cell biology research), revealed that BAF60c significantly enhances the interaction between these transcription factors and Brg1, facilitating chromatin remodeling. Additionally, studies have found that the expression of Nppa (atrial natriuretic factor) is reduced in Smarcd3 (BAF60c) knockdown mouse embryos (Lickert et al., 2004). This suggests that BAF60c plays a crucial role in the regulation of cardiac gene expression. Similarly, in *Xenopus* embryos, BAF60c promoted transcriptional synergy between these transcription factors and Brg1 during early heart development (Gallagher et al., 2012). In addition to cooperating with classical cardiac factors, BAF60c also directly interacts with myocardin (MYOCD), as confirmed by *in vitro* binding assays, and regulates contractile, metabolic, and sarcomeric gene programs critical for heart function (Sun et al., 2018). Interestingly, in c-kit^+^ cardiac progenitor cells, BAF60c overexpression failed to enhance—and even inhibited—the transcriptional activity of GATA4 and TBX5, suggesting a dosage-sensitive effect on cardiac gene regulation (Al-Maqtari et al., 2017).

## Regulation of BAF60c complex in cardiac development and differentiation

BAF60c is also regulated by various factors to ensure normal heart development and function. ISL LIM Homeobox 1 (ISL1) acts as an upstream positive regulator of BAF60c, and cooperates with the Brg1-BAF60c complex to promote the differentiation of cardiac progenitor cells into cardiomyocytes (Gao et al., 2019). Klaus et al. (2012) found that the Wnt Signaling Pathway/Beta-Catenin Pathway (Wnt/β-catenin) activates cardiac-specific transcription factors, including BAF60c, Nkx2.5, and Isl1, which are essential for the differentiation of cardiac progenitor cells into cardiomyocytes. The secreted protein cerberus-1 (Cer1) indirectly induces the expression of BAF60c and cardiac-specific transcription factors, such as GATA4 and TBX5, by inhibiting the Nodal signaling pathway (Nodal signaling) and the bone morphogenetic protein pathway (BMP signaling), thereby promoting cardiac gene activation and cardiomyocyte differentiation (Cai et al., 2013). In embryonic stem cell studies, the G-protein–coupled Apelin receptor (Apj) was shown to be expressed in second heart field mesodermal cells. Apj activates Cerberus-1, thereby promoting Baf60c expression and facilitating cardiomyocyte differentiation (D'Aniello et al., 2013). Researchers also used lentiviral transduction to introduce GATA4, Myocyte Enhancer Factor 2C (MEF2C), NKX2.5, TBX5, and BAF60c into human c-kit^+^ cardiac progenitor cells. During heart development, Cerberus like 2 (Cerl2) inhibits the Transforming Growth Factor Beta (TGFβ)/Nodal signaling pathway, reducing BAF60c expression (Araújo et al., 2014). In *Cerl2* knockout mouse embryos, BAF60c levels are significantly elevated, leading to left ventricular hyperplasia and increased cardiomyocyte proliferation (Araújo et al., 2014) (Table 3). In addition to transcriptional regulation, BAF60c also functions at the chromatin level through complex recruitment mechanisms in the heart. It directly interacts with cardiac transcription factors such as GATA4, NKX2.5, TBX5, and MYOCD to facilitate the assembly of the SWI/SNF complex at cardiac gene loci, thereby promoting sarcomere formation, cardiac metabolism, and myocardial differentiation (Sun et al., 2018). While post-translational modifications (PTMs) of BAF60c such as phosphorylation have been described in skeletal muscle and metabolic tissues, their roles in the heart remain to be fully elucidated.

## The cooperative role of BAF60 subunits in cardiac differentiation and development

Studies on pluripotent ESCs have revealed that BAF60a, BAF60b, and BAF60c are dynamically expressed during cardiogenesis and contribute to distinct but coordinated roles in heart development (Hota et al., 2019). BAF60a is enriched in ESCs and regulates pluripotency and early differentiation. As differentiation progresses, BAF60c expression increases and replaces BAF60a, enabling the formation of cardiac-specific SWI/SNF complexes that activate sarcomeric and metabolic gene programs. BAF60b functions mainly in cardiac progenitor cells, collaborating with Brg1 to initiate cardiac gene expression. Additionally, BAF60c cooperates with other SWI/SNF subunits like BAF170 (a component of the SWI/SNF chromatin remodeling complex that plays a crucial role in gene regulation) to ensure cardiac-specific gene expression and morphogenesis (Hota et al., 2019). BAF60a also contributes to the migration and polarity of cardiac progenitor cells by interacting with the transcription factor TBX1, enhancing its occupancy at the *Wnt5a* (*Wingless-type MMTV integration site family, member 5A*) promoter, and promoting H3K4 monomethylation-mediated chromatin activation (Chen et al., 2012). Wnt5a activation facilitates key morphogenetic processes necessary for proper cardiac structure formation and the prevention of congenital heart defects (Chen et al., 2012). Together, these findings suggest that BAF60 isoforms cooperate through temporal replacement, subunit composition switching, and transcription factor–specific interactions, enabling the SWI/SNF complex to drive stage-specific transcriptional programs from pluripotency to terminal cardiac differentiation (Table 3).

## Multifactorial regulation of BAF60c-driven cardiac fate conversion of non-cardiomyocytes

Under specific conditions, BAF60c cooperates with key cardiac transcription factors—primarily GATA4, TBX5, and NKX2.5—to promote the transdifferentiation of non-cardiac cells into beating cardiomyocytes. This combination has been shown to activate cardiac gene expression and repress non-cardiac mesodermal programs in various contexts, including mouse mesoderm, human embryonic stem cells (hESCs), and adipose-derived mesenchymal stem cells (ADMSCs) (Takeuchi and Bruneau, 2009; Dixon et al., 2011; Li et al., 2015). Furthermore, overexpression of BAF60c together with GATA4 and Mesoderm Posterior BHLH Transcription Factor 1 (MESP1) via transient plasmids has been reported to activate cardiac genes, thereby promoting the differentiation of human pluripotent stem cells (hPSCs) into cardiomyocyte-like cells (Hartung et al., 2013). Lalit et al. (2017) demonstrated that lentiviral transduction of Mesp1, TBX5, GATA4, Nkx2.5, and Baf60c (MTGNB) activates the Wnt/β-catenin signaling pathway and the Janus Kinase/Signal Transducer and Activator of Transcription signaling pathway in mouse fibroblasts, reprogramming them into induced cardiac progenitor cells (iCPCs). Similarly, Narita et al. (2022) showed that the combination of six transcription factors—Gata6, GATA4, Mef2a, Baf60c, Klf15, and Myocd—delivered via lentiviral gene transfer into adult adipose-derived regenerative cells (ADRCs), effectively induced cardiac gene expression. Although these reprogrammed ADRCs (6F-ADRCs) exhibited multiple cardiac genes and improved cardiac function when transplanted into an acute myocardial infarction animal model, mature sarcomere structures and spontaneous beating cardiomyocytes were not observed (Narita et al., 2022). This suggests that while BAF60c contributes to cardiac gene activation, further refinements are required to achieve mature cardiomyocyte formation (Narita et al., 2022). These studies highlighted the critical role of BAF60c in cardiac gene activation and its potential for cardiac regenerative medicine (Table 4).

**TABLE 4:** Roles of BAF60c Subunits in cardiac reprogramming.

Muscle type	Reprogramming		
	Cell type	Transcription factors	Outcome
Cardiac muscle	mouse mesoderm (Takeuchi and Bruneau, 2009)	BAF60C + GATA4 +TBX5 (Takeuchi and Bruneau, 2009)	Beating cardiomyocytes (Takeuchi and Bruneau, 2009)
	hESCs (Dixon et al., 2011)	BAF60C + GATA4+NKX2.5+TBX5 (Dixon et al., 2011)	Beating cardiomyocytes (Dixon et al., 2011)
	ADMSCs (Li et al., 2015)	BAF60C + GATA4+TBX5 (Li et al., 2015)	Beating cardiomyocytes (Li et al., 2015)
	hPSCs (Hartung et al., 2013)	BAF60C + GATA4+MESP1 (Hartung et al., 2013)	Cardiomyocyte-like cells (Hartung et al., 2013)
	Mouse fibroblasts (Lalit et al., 2017)	BAF60C + GATA4+NKX2.5+TBX5+MESP1 (Lalit et al., 2017)	iCPCs (Lalit et al., 2017)
	ADRCs (Narita et al., 2022)	BAF60C + GATA4+MEF2A + KIF15+MYOCD + GATA6 (Narita et al., 2022)	Exhibited multiple cardiac genes (Narita et al., 2022)

This table lists various cell types reprogrammed into cardiomyocyte-like cells through the ectopic expression of BAF60c and cardiac transcription factors. References in square brackets correspond to studies supporting the respective findings and mechanisms.

hESCs, Human Embryonic Stem Cells; ADMSCs, Adipose-Derived Mesenchymal Stem Cells; hPSCs, Human Pluripotent Stem Cells; ADRCs, Adult Adipose-Derived Regenerative Cells; iCPCs, Induced Cardiac Progenitor Cells; MESP1, Mesoderm Posterior BHLH, Transcription Factor 1.

## BAF60c mediates cardiac hypertrophy and heart failure

**FIGURE 2:**
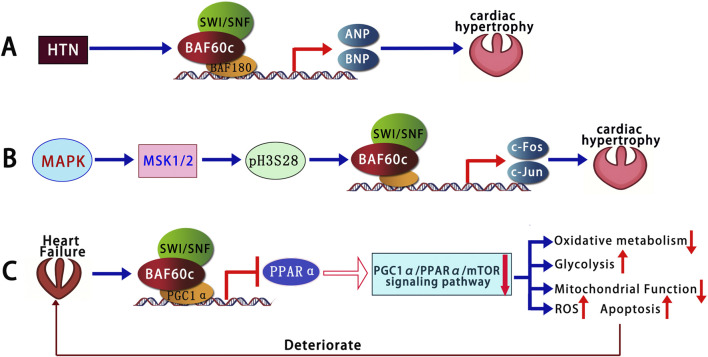
BAF60c-mediated chromatin remodeling in cardiac hypertrophy and heart failure. **(A)** In hypertensive hearts, SWI/SNF subunits including BAF60c are upregulated and activate fetal cardiac genes. **(B)** MAPK–MSK signaling induces H3S28 phosphorylation, facilitating recruitment of SWI/SNF and activation of immediate early genes. **(C)** In heart failure, elevated BAF60c suppresses oxidative metabolism via PGC1α/PPARα/mTOR inhibition, promoting glycolysis and cardiomyocyte apoptosis. SWI/SNF, SWItch/Sucrose Non-Fermentable; MAPK, Mitogen-Activated Protein Kinase; MSK, Mitogen- and Stress-activated Kinase; H3S28, Histone H3 Serine 28; ANP, Atrial Natriuretic Peptide; BNP, Brain Natriuretic Peptide; PGC1α, Peroxisome Proliferator-Activated Receptor Gamma Coactivator 1-Alpha; PPARα, Peroxisome Proliferator-Activated Receptor Alpha; mTOR, Mechanistic Target of Rapamycin; ROS, Reactive Oxygen Species; HTN, Hypertension; c-Fos, FBJ murine osteosarcoma viral oncogene homolog; c-Jun, Jun proto-oncogene.

BAF60c is a critical regulator of cardiac metabolic adaptation, promoting glycolytic flux and mitochondrial biogenesis, particularly under stress conditions such as hypertrophy or heart failure. In a rat model of salt-induced cardiac hypertrophy, the expression levels of several SWI/SNF chromatin remodeling subunits—including Brg1, Baf180, and BAF60c—were significantly elevated in cardiac tissues (Mehrotra et al., 2013). These subunits were recruited to the promoters of fetal cardiac genes, such as Atrial Natriuretic Peptide (ANP) and Brain Natriuretic Peptide (BNP), which was accompanied by increased chromatin accessibility and histone modification changes, indicating a highly active transcriptional state. These findings suggest that SWI/SNF subunits, particularly BAF60c, regulate cardiac gene expression through chromatin remodeling, mediating salt-induced cardiac hypertrophy (Mehrotra et al., 2013) (Figure 2A).

During cardiac hypertrophy, the MAPK signaling pathway activates mitogen- and stress-activated protein kinase (MSK), which leads to the phosphorylation of Histone H3 Serine 28 (H3S28). This modification subsequently recruits the Brg1 subunit of the BAF60 complex, specifically BAF60c. This activation induces the expression of immediate early genes, such as *c-Fos* and *c-Jun* (Robinson et al., 2022). These genes encode products that form key components of the activator protein-1 (AP-1) complex. This complex rapidly initiates cellular responses to external stress by regulating protein synthesis, cell growth, and metabolic processes, ultimately promoting cardiomyocyte enlargement and contributing to cardiac hypertrophy (Robinson et al., 2022) (Figure 2B).

Chen Q. et al. (2022) further investigated that BAF60c expression was significantly increased in adult Heart Failure (HF) rats, resulting in an imbalance between oxidative metabolism and glycolysis, as well as impaired mitochondrial function. Knockdown of BAF60c in HF rats restored oxidative metabolism, improved glucose uptake, enhanced mitochondrial function, and reduced apoptosis by activating the Peroxisome Proliferator-Activated Receptor Gamma Coactivator 1-Alpha/Peroxisome Proliferator-Activated Receptor Alpha/Mechanistic Target of Rapamycin (PGC1α/PPARα/mTOR) signaling pathway. *In vitro* experiments using H9C2 cells (derived from rat heart and commonly used to study cardiomyocyte differentiation and cardiac biology) treated with isoproterenol (ISO) (a synthetic β-adrenergic receptor agonist used in cardiac research to study heart function and induce experimental cardiac stress) confirmed these findings, showing that BAF60c knockdown increased oxidative metabolism-related markers, promoted cell proliferation, improved ATP levels, and reduced apoptosis (Chen Q. et al., 2022) (Figure 2C). These results suggest that BAF60c downregulation could be beneficial for maintaining myocardial energy metabolism and improving cell survival under HF conditions, providing new insights into potential therapeutic strategies for heart failure (Chen Q. et al., 2022).

## BAF60 isoforms in smooth muscle: context and importance

Smooth muscle cells (SMCs) play essential roles in regulating vascular tone, blood pressure, and organ contractility, and their phenotypic plasticity is central to the pathogenesis of vascular diseases (Sohni et al., 2012; Zhao et al., 2022). Unlike skeletal and cardiac muscle, which are terminally differentiated and contract rhythmically, SMCs retain the ability to switch between a contractile and a synthetic phenotype in response to environmental cues (Zhao et al., 2022; Chang et al., 2020). This dynamic behavior is tightly controlled by transcriptional and epigenetic mechanisms, including chromatin remodeling (Sohni et al., 2012; Zhao et al., 2022). While studies of BAF60 isoforms in skeletal and cardiac muscle have been extensively conducted, their roles in smooth muscle biology have only recently begun to emerge. Notably, recent research has demonstrated distinct and even opposing functions of BAF60c and BAF60a in SMC differentiation, vascular homeostasis, and the development of diseases such as abdominal aortic aneurysm (AAA) and atherosclerosis (Zhao et al., 2022; Chang et al., 2020). These findings expand our understanding of the BAF60 subunits beyond striated muscle and underscore their relevance in vascular pathophysiology.

## Opposing roles of BAF60c and BAF60a in smooth muscle homeostasis and vascular diseases

BAF60c has been shown to play a crucial regulatory role in the differentiation of multipotent adult progenitor cells (MAPCs) into smooth muscle cells (SMCs) (Sohni et al., 2012). Transforming Growth Factor Beta 1 (TGFβ1) activates the SMAD2/3 complex through its receptor, which then translocates into the nucleus and binds to SMAD-binding elements (SBEs) within the BAF60c promoter, driving the transcription and expression of BAF60c. BAF60c interacts with SRF to form a complex that binds to CArG box regions, co-regulating the expression of smooth muscle-specific genes such as *a-SMA* and *SM22a*, thereby promoting smooth muscle cell differentiation. Overexpression of BAF60c was sufficient to induce the differentiation of rat MAPCs into SMCs even in the absence of exogenous cytokines. However, the resulting cells exhibited an immature phenotype, characterized by increased proliferation and reduced expression of contractile markers (Sohni et al., 2012).

**FIGURE 3:**
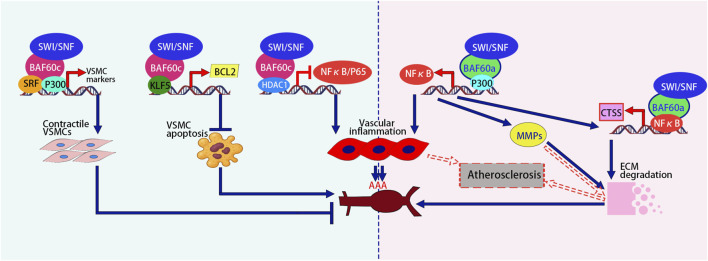
Opposing roles of BAF60c promotes the contractile phenotype, inhibits apoptosis via KLF5-BCL2, and reduces inflammation via HDAC1-mediated NF-κB suppression, thereby protecting against AAA. In contrast, BAF60a activates NF-κB, promotes vascular inflammation, upregulates MMPs and CTSS, enhances ECM degradation, and contributes to AAA and atherosclerosis progression. The figure is based on illustrations from Zhao et al. (2022) and includes content derived from Chang et al. (2020) and Jiang et al. (2022). SRF, Serum Response Factor; ECM, Extracellular Matrix; NF-κB, Nuclear Factor-kappa B; MMPs, Matrix Metalloproteinases; CTSS, Cathepsin S; AAA, Abdominal Aortic Aneurysm.

In addition to promoting smooth muscle differentiation, research (Zhao et al., 2022) has shown that BAF60c plays a protective role in preventing AAA formation by maintaining vascular smooth muscle cell (VSMC) homeostasis through epigenetic mechanisms. In human and mouse AAA tissues, the expression of VSMC BAF60c is significantly reduced. Mice with VSMC-specific knockout of the *Baf60c* gene exhibit a higher incidence of AAA, accompanied by enhanced elastin degradation, inflammatory cell infiltration, VSMC phenotype switch, and apoptosis. Mechanistic studies indicate that BAF60c interacts with SRF and its coactivator P300, enhancing the binding of the SWI/SNF complex to the promoters of Vascular Smooth Muscle Cell (*VSMC*)-specific genes, thereby promoting the transcriptional activity of contraction-related genes and maintaining the contractile phenotype of VSMCs. Additionally, BAF60c interacts with Histone Deacetylase 1 (HDAC1) to inhibit the chromatin opening of the Nuclear Factor-kappa B p65 subunit (NF-κB/p65), thereby preventing the transcriptional activation of inflammation-related genes in VSMCs. Furthermore, BAF60c promotes the binding of Kruppel-like Factor 5 (KLF5) to the B-cell lymphoma 2 (BCL2) promoter, enhancing BCL2 transcription and thereby inhibiting VSMC apoptosis (Zhao et al., 2022) (Figure 3).

Opposite to BAF60c, BAF60a plays a promotive role in the formation of AAA (Chang et al., 2020). The expression of BAF60a is significantly increased in both human and mouse AAA lesion tissues. In two mouse models (Angiotensin II-induced model and elastase-induced model), the specific knockout of BAF60a in VSMCs significantly reduced the incidence of AAA, while also reducing vascular inflammation, monocyte infiltration, elastin degradation, and extracellular matrix (ECM) degradation. Mechanistic studies revealed that BAF60a enhances the transcriptional activity of NF-κB through its interaction with p300, thereby triggering inflammatory responses. Furthermore, NF-κB enhances the transcription and expression of its target gene, matrix metalloproteinases (*MMPs*), thereby promoting the degradation of the ECM. In addition, BAF60a and NF-κB synergistically increased the expression of cathepsin S (CTSS), which, through ECM degradation, further exacerbated the fragility of the aortic wall. These results suggest that BAF60a promotes AAA formation by facilitating inflammation and ECM degradation, and therefore, BAF60a may serve as a potential target for the prevention and treatment of AAA (Chang et al., 2020) (Figure 3).

Jiang et al. (2022) encapsulated BAF60a siRNA in galactose-modified chitosan nanoparticles (GTANPs) and intravenously delivered them to Apolipoprotein E (ApoE) knockout mice, a widely used model for studying atherosclerosis and lipid metabolism. The treatment with GTANPs/siBaf60a significantly reduced plasma cholesterol levels and atherosclerotic plaque area by suppressing inflammation and ECM degradation. These findings are particularly important because BAF60a promotes pro-inflammatory gene expression and matrix breakdown in vascular tissue, processes that accelerate plaque formation and destabilization. Targeted silencing of BAF60a in ApoE-KO mice highlights its potential as a therapeutic target in atherosclerosis, offering a novel strategy to simultaneously modulate lipid metabolism and vascular inflammation for plaque stabilization and regression. Mechanistic studies revealed that BAF60a promotes vascular wall inflammation by upregulating pro-inflammatory cytokines (such as MCP-1, TNF-α, etc.) and modulating macrophage polarization. Meanwhile, BAF60a enhances the expression of MMPs, which promotes the degradation of the ECM, destabilizing the vascular wall and exacerbating the pathological progression of atherosclerosis (Jiang et al., 2022) (Figure 3; Table 5). These findings highlight the opposing roles of BAF60a and BAF60c in vascular diseases such as AAA and atherosclerosis, suggesting that their targeted modulation could offer novel therapeutic strategies with significant clinical potential.

**TABLE 5:** Opposing roles of BAF60c and BAF60a in smooth muscle.

Muscle type	Complex condition	Binding regions	Cell types	Upstream factors		BAF60 roles
				Upregulation	Downregulation	
Smooth muscle	BAF60c + SRF + BRG1 (Sohni et al., 2012)	CArG box (Sohni et al., 2012)	MAPCs (Sohni et al., 2012)	TGFβ1 (by activating SMAD2/3) (Sohni et al., 2012)	N/A	Opposing Roles: BAF60c maintains contractile phenotype, suppresses inflammation and apoptosis to prevent AAA formation; BAF60a enhances ECM degradation and inflammation, to promote AAA formation

This table contrasts the protective role of BAF60c with the pathological effects of BAF60a in smooth muscle homeostasis and vascular diseases. References in square brackets correspond to studies supporting the respective findings and mechanisms.

MAPCs, Multipotent Adult Progenitor Cells; TGFβ1, Transforming Growth Factor Beta 1; AAA, abdominal aortic aneurysm; ECM, Extracellular Matrix; SRF, serum response factor; N/A, not available or not applicable; NF-κB, Nuclear Factor-kappa B.

## Discussion

The BAF60 subunit, an essential component of the SWI/SNF complex, mediates chromatin remodeling, a critical epigenetic mechanism regulating gene expression across skeletal muscle, cardiac muscle, and smooth muscle. In skeletal muscle, BAF60c collaborates with MyoD to regulate muscle-specific gene expression, driving differentiation and development. It promotes glycolytic metabolism and interacts with metabolic factors to support energy adaptation, playing a crucial role in muscle regeneration. Additionally, BAF60c facilitates skeletal muscle reprogramming by enhancing chromatin accessibility at myogenic loci, enabling the conversion of non-muscle cells into myogenic lineages. In contrast, BAF60a and BAF60b antagonize BAF60c by suppressing differentiation, highlighting their regulatory balance in skeletal muscle biology.

In cardiac muscle, BAF60c interacts with transcription factors such as Nkx2.5, GATA4, and TBX5 to activate cardiac-specific genes, promoting heart differentiation and development. Furthermore, BAF60c cooperates with core transcription factors to drive the cardiac fate conversion of non-cardiomyocytes, thereby participating in cardiac reprogramming and offering a potential therapeutic strategy for cardiac regeneration. BAF60c is implicated in hypertension-induced cardiac remodeling. During heart failure, BAF60c drives a metabolic shift characterized by increased glycolytic activity, mitochondrial dysfunction, and cardiomyocyte apoptosis. Targeted downregulation of BAF60c may offer a promising therapeutic strategy for heart failure and remodeling. BAF60a and BAF60b complement BAF60c at specific developmental stages, demonstrating their collaborative roles in cardiac muscle biology. In smooth muscle, BAF60c interacts with SRF to promote smooth muscle cell differentiation. Additionally, BAF60c maintains homeostasis by maintaining the contractile phenotype, reducing inflammation and preventing apoptosis, which protects against vascular pathologies such as AAA. Conversely, BAF60a promotes inflammation and ECM degradation, contributing to the progression of AAA and atherosclerosis.

Although BAF60c functions across all muscle types as a cofactor for lineage-specific transcription factors, its binding partners and transcriptional targets vary, conferring tissue specificity. In skeletal muscle, it cooperates with MyoD; in cardiac muscle, with NKX2.5, GATA4, and TBX5; and in smooth muscle, with SRF and p300. These interactions reflect both shared and distinct mechanisms shaped by chromatin context and competing BAF60 isoforms. While BAF60c generally promotes differentiation, the outcome—myogenesis, cardiogenesis, or vasoprotection—is context-dependent. In contrast, BAF60a antagonizes differentiation in skeletal and smooth muscle but plays supportive roles in the heart. These differences highlight the need to define isoform-specific interactomes to fully understand BAF60-mediated regulation in muscle.

Future studies should further elucidate the dynamic regulatory mechanisms of BAF60 isoforms in muscle differentiation, regeneration, reprogramming, and metabolic homeostasis across skeletal, cardiac, and smooth muscle. In particular, determining the precise molecular determinants underlying the antagonistic, synergistic, or redundant roles of BAF60a, BAF60b, and BAF60c in specific muscle lineages and physiological or pathological contexts remains a critical and largely unexplored area. Addressing this knowledge gap may uncover new therapeutic opportunities to selectively modulate BAF60 subunit activity in muscle-related metabolic and vascular diseases.

While most conclusions in this review are drawn from studies using cell lines (e.g., C2C12, ESCs, iPSCs) and transgenic or knockout mouse models, these systems have inherent limitations. *In vitro* models may lack physiological complexity, and murine models differ from humans in gene regulation and metabolism. These factors should be considered when interpreting findings and highlight the need for complementary validation in human tissues or advanced models.
